# A Large Retroperitoneal Liposarcoma Mimicking an Angiomyolipoma on Pre-operative Imaging: A Case Report

**DOI:** 10.7759/cureus.44325

**Published:** 2023-08-29

**Authors:** Daniela Usuga, Elias Atri, Ferial Alloush, Robert Poppiti, Akshay Bhandari

**Affiliations:** 1 Medical Student, Florida International University, Herbert Wertheim College of Medicine, Miami, USA; 2 Urology, Mount Sinai Medical Center, Miami Beach, USA; 3 Pathology, Mount Sinai Medical Center, Miami Beach, USA

**Keywords:** soft-tissue sarcoma, well-differentiated liposarcoma, retroperitoneal adrenal mass, renal angiomyolipoma, retroperitoneal liposarcoma

## Abstract

Retroperitoneal masses present a diagnostic challenge due to their elusive origin and varied clinical manifestations. Among these masses, retroperitoneal liposarcomas, rare tumors of mesenchymal origin, often grow asymptomatically until compressing surrounding structures, necessitating accurate and early diagnosis. Renal angiomyolipomas (AMLs) have also been reported to mimic retroperitoneal liposarcomas on radiographic imaging, further complicating diagnostic processes.

The presented case report describes a rare instance of a large well-differentiated liposarcoma that mimicked a renal angiomyolipoma on imaging in a 58-year-old male patient. The patient initially presented with worsening abdominal distension, early satiety, and left-sided flank pain for the past year. Radiographic imaging revealed a large mixed echogenic lesion measuring 22 x 13 cm in the left kidney with diffuse fat contribution, suspected to be a giant renal angiomyolipoma. The patient underwent selective arterial embolization by interventional radiology. Follow-up imaging eight months later showed an increase in the size of the mass, raising suspicion of a liposarcoma. Surgical resection of the mass and a radical left nephrectomy were performed, with final pathology confirming the diagnosis of a well-differentiated liposarcoma. This case highlights the importance of accurate diagnosis and the potential for liposarcomas to mimic other masses on imaging, despite their rarity.

## Introduction

Retroperitoneal masses, which arise in the retroperitoneum but do not originate from retroperitoneal organs, can be reactive or neoplastic. Due to their location, diagnosing these masses by imaging can be challenging, increasing the importance of being aware of the characteristic imaging features and clinical information to generate an accurate differential diagnosis [1]. Primary retroperitoneal masses are characterized on imaging by ensuring the mass is in the retroperitoneum and not originating from another organ. Diagnostic features that can be noted in imaging, such as magnetic resonance imaging (MRI) and computerized tomography (CT) include composition, size, enhancements, and relationships with adjacent structures [1]. The clinical presentation of retroperitoneal masses can be extremely variable, adding to the difficulty of diagnosis. The final diagnosis can only be confirmed by gross and histopathologic examination [2].

Retroperitoneal liposarcomas, rare tumors of mesenchymal origin, can grow asymptomatically until they begin to compress surrounding structures such as kidneys, ureters, lymphatic drainage, and other abdominal organs. They are commonly underdiagnosed due to the lack of symptoms and the rarity of such tumors [3]. In the literature, there is a case of a renal angiomyolipoma (AML) mimicking a retroperitoneal well-differentiated liposarcoma (WDLs) due to similarities in radiographic imaging [3,4]. Correctly diagnosing AML and WDLs is important as they have different treatment modalities. AMLs have multiple treatment options, including active surveillance, selective arterial embolization (SAE), ablative therapies, mammalian target rapamycin (mTOR) inhibitors, and surgery. In contrast, WDLs have limited therapeutic options, with surgery as the mainstay of therapy.

In the literature, there are a few prior cases of a giant extrarenal retroperitoneal angiomyolipoma that mimicked a liposarcoma [5,6,7]. Although the presented case is similar in that a renal angiomyolipoma mimicked a liposarcoma on imaging, the size of the mass fell short of being classified as a giant, as it weighed 14 kg. In some literature, liposarcoma size has been classified as "giant" if the weight is over 20 kg, while those under 20 kg are usually termed large liposarcomas [5]. The objective of this study is to report a case of a large well-differentiated liposarcoma that mimicked a renal angiomyolipoma (AML) on imaging and underwent SAE prior to surgical resection.

## Case presentation

**Figure 1:**
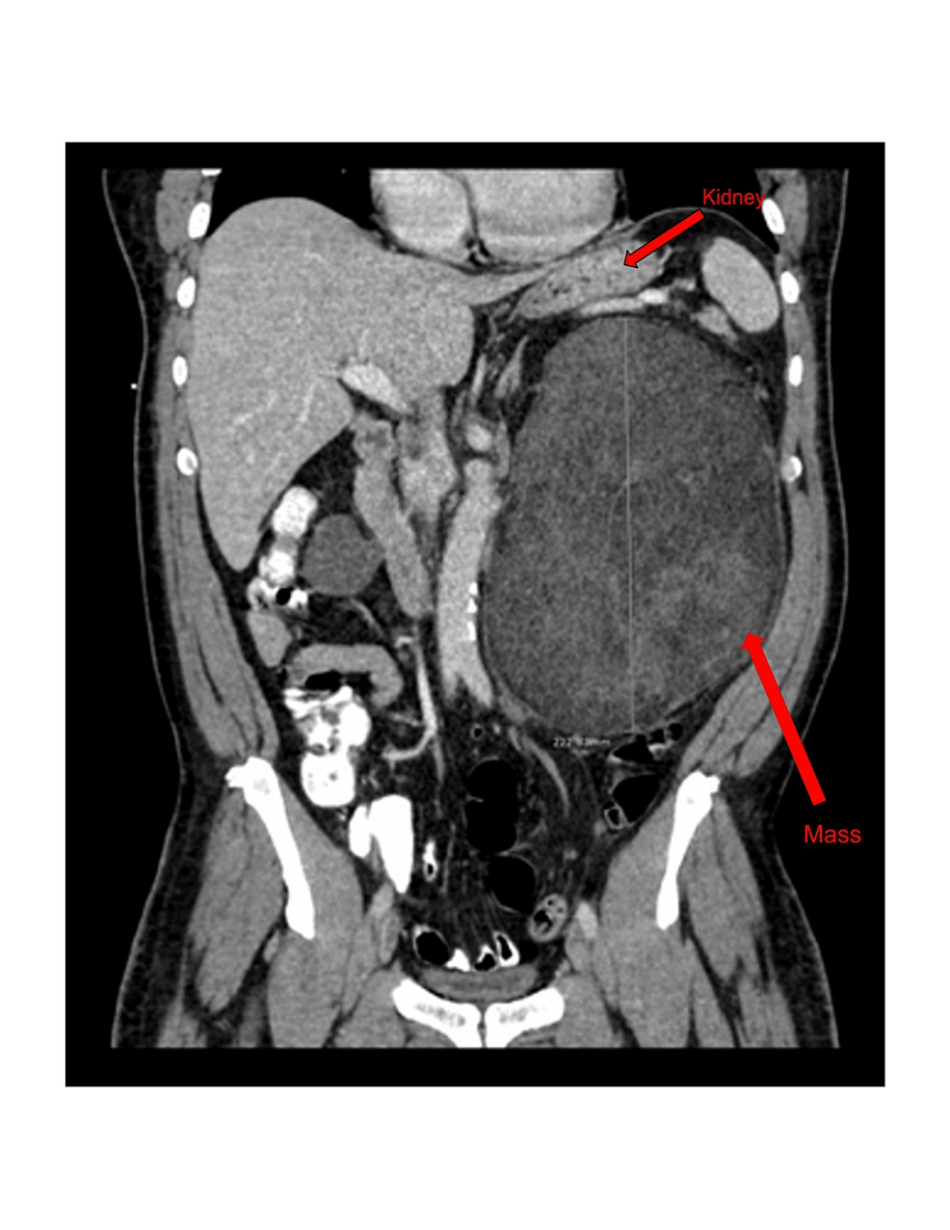
Patient imaging CT with IV contrast of abdomen in coronal view. Large mixed echogenic lesion measuring 22 x 13 cm in size with diffuse fat contribution and soft tissue elements suspicious for giant renal angiomyolipoma with mass effect but no definitive acute hemorrhage CT: computerized tomography; IV: intravenous

**Figure 2:**
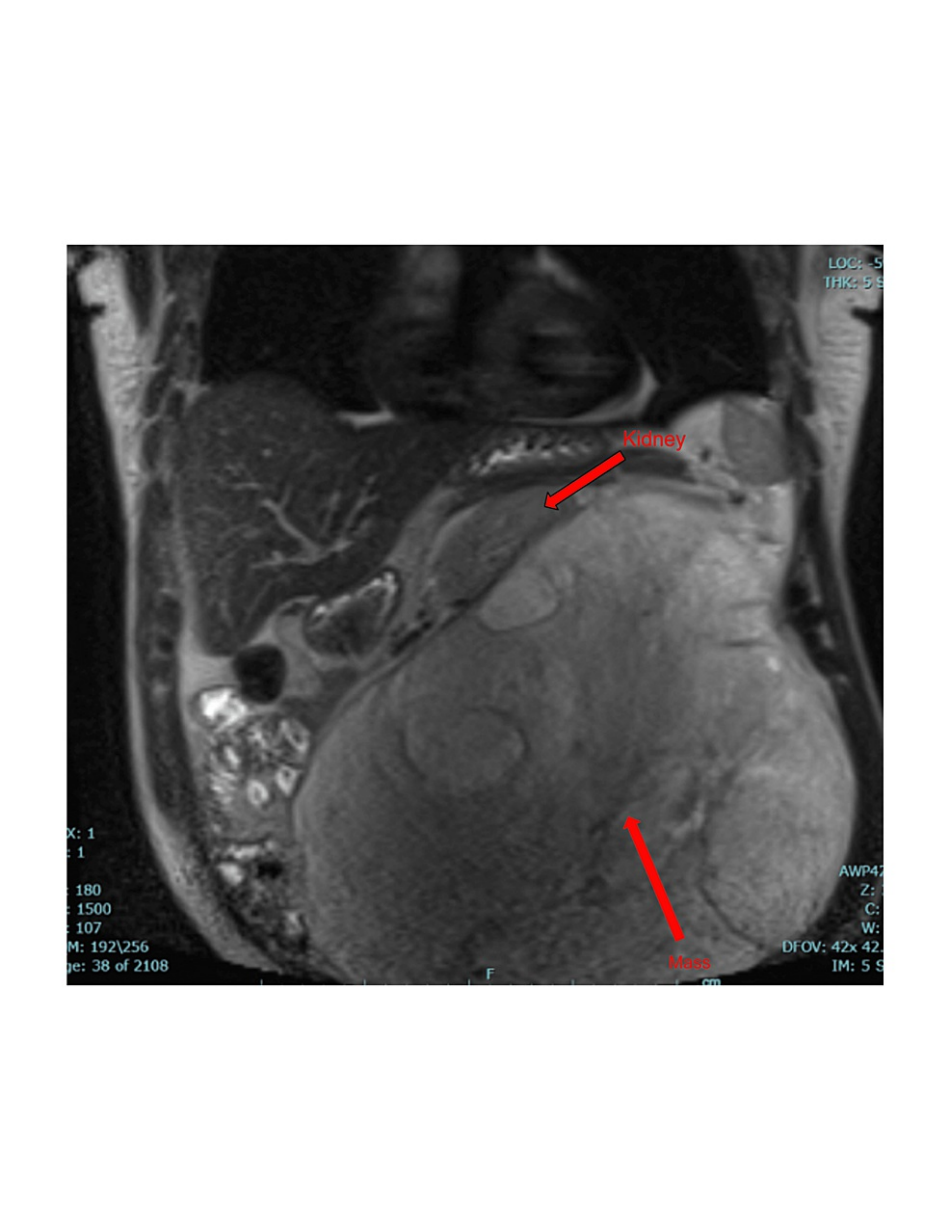
MRI of abdomen status post embolization of left retroperitoneal fat-containing mass displacing the left kidney MRI: Magnetic Resonance Imaging

A 58-year-old African American male presented to the urology office with a chief complaint of abdominal distention, early satiety, abdominal and left-sided flank pain that was progressively worsening for the past year. The patient denied any gross hematuria or dysuria, but endorsed 12 month history of early satiety, abdominal pain, and significant weight gain. His past medical history, family history, and surgical history had no significant relevance to the case. The patient had previously presented to emergency department (ED) for abdominal pain, and was thought to have diverticulitis in the sigmoid colon. On physical exam, patient was noted to have abdominal distention with a large palpable left sided mass. He had an abdominal CT with intravenous (IV) contrast, which found hypodensities along the lateral margin of the right kidney measuring 1.5 cm in size, thought to represent cysts. Adjacent to the left kidney, a large mixed echogenic lesion measuring 22 x 13 cm with diffuse fat contribution and soft tissue elements, suspicious for a giant renal AML with mass effect was discovered, however no definitive acute hemorrhage or enhancing blood vessels were identified (Figure 1). The patient underwent SAE by interventional radiology in an attempt to reduce the tumor size prior to seeking a urology consult. An MRI of the abdomen taken eight months later reported an interval increase in the size of the left retroperitoneal fat-containing mass, which was now measuring 20 x 18.6 x 22.2 cm and compressing the inferior vena cava (Figure 2). Radiology suggested that although the findings were consistent with the history of a left renal angiomyolipoma, other neoplastic processes such as liposarcoma should be considered in the differential.

**Figure 3:**
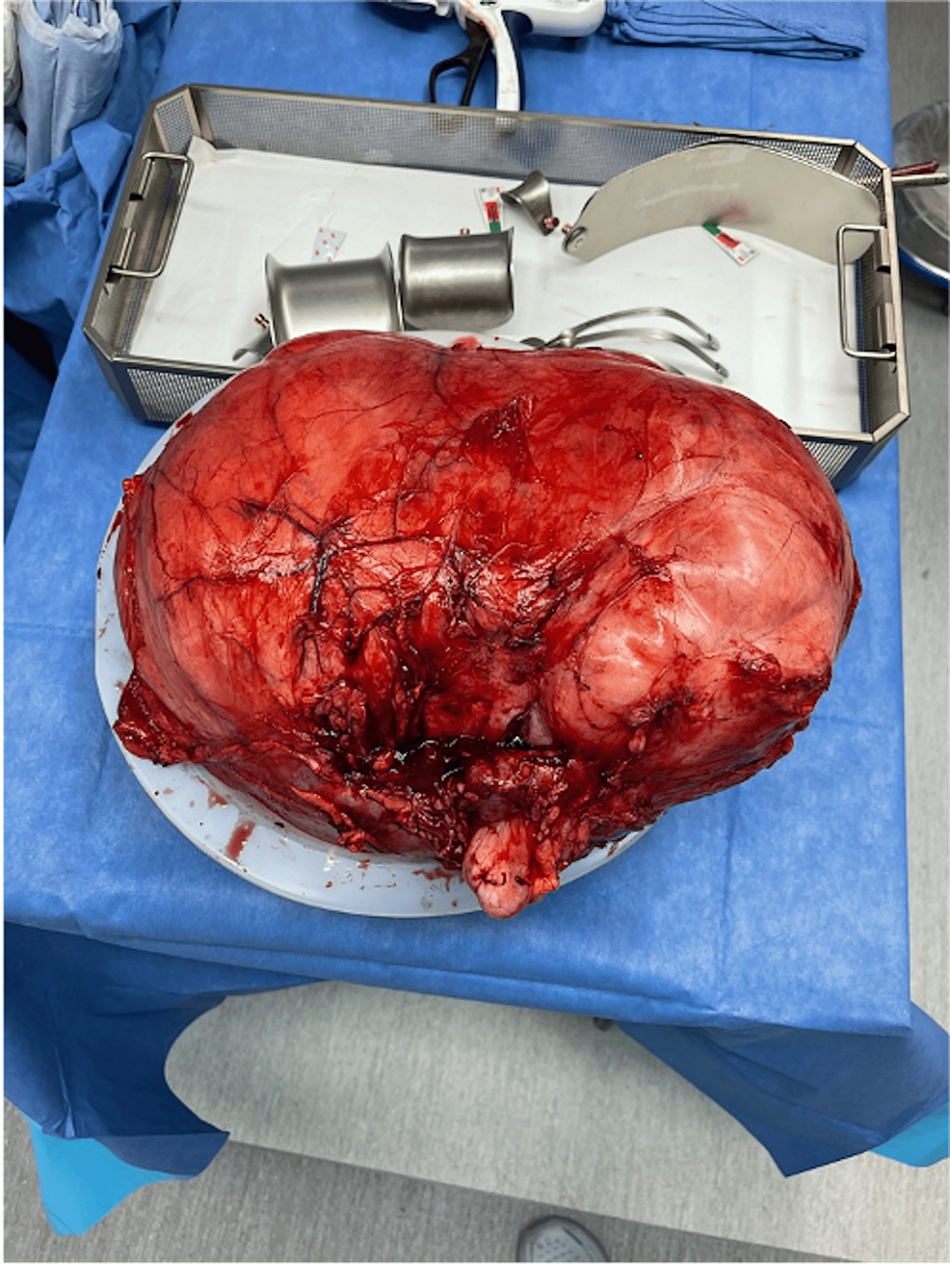
Gross specimen measured 39 x 32 x 22 cm and weighed 14,360g (14kg)

During the physical exam, the patient's abdomen was found to be distended, and a large left flank mass was palpated. No other abnormalities were noted on the physical exam. After reviewing the clinical history and serial imaging, there was concern that this mass could be a liposarcoma. Therefore, it was recommended that the mass be resected along with a radical left nephrectomy due to the location of the mass. The mass was removed with 2 cm surgical margins. The final pathology report stated that the tan-yellow, lobulated mass measured 39x32x 22 cm and weighed 14,360 g (14kg). The external surface of the tumor is tan-yellow, smooth, and glistening and was resected intact. The histologic type of the tumor was determined to be a WDL histologic grade 1 (Figure 3).

**Figure 4:**
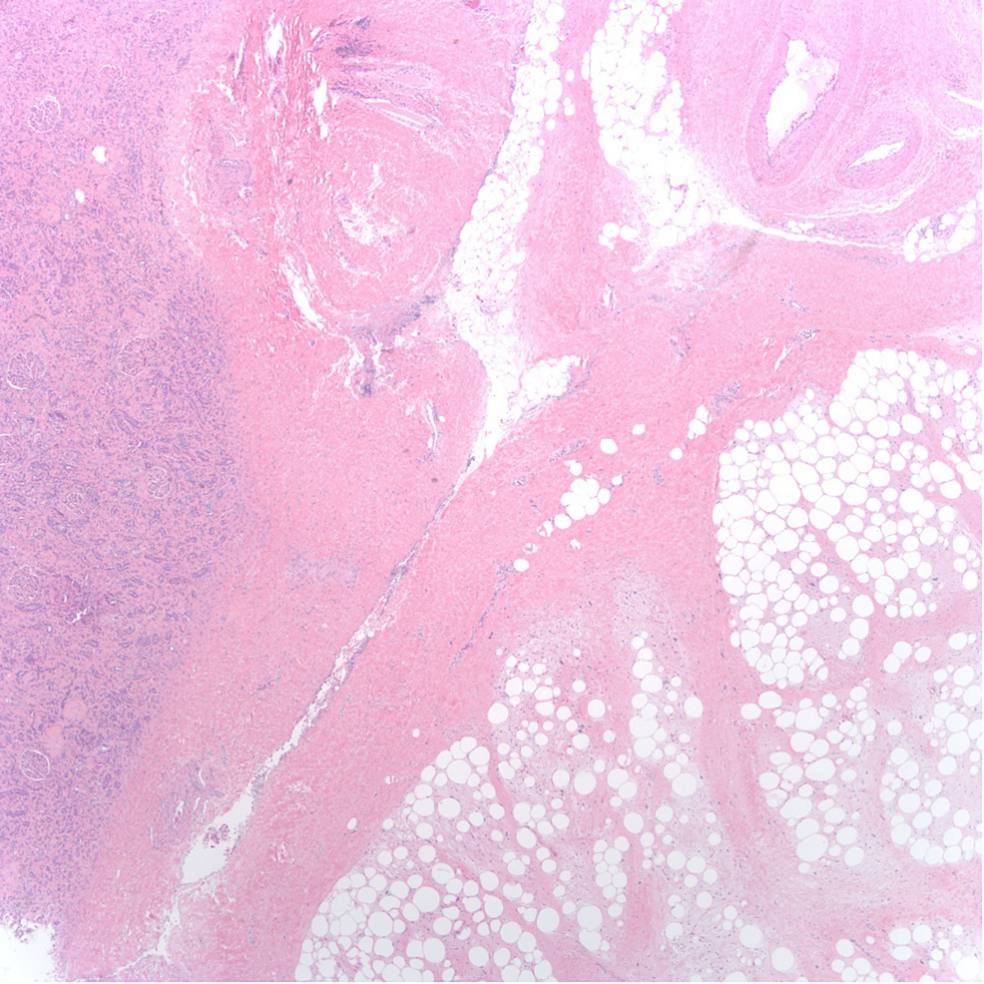
25x magnification H&E stain microscopic image showing well-differentiated liposarcoma on the right and kidney tissue on the left. H&E: Hematoxylin and Eosin

**Figure 5:**
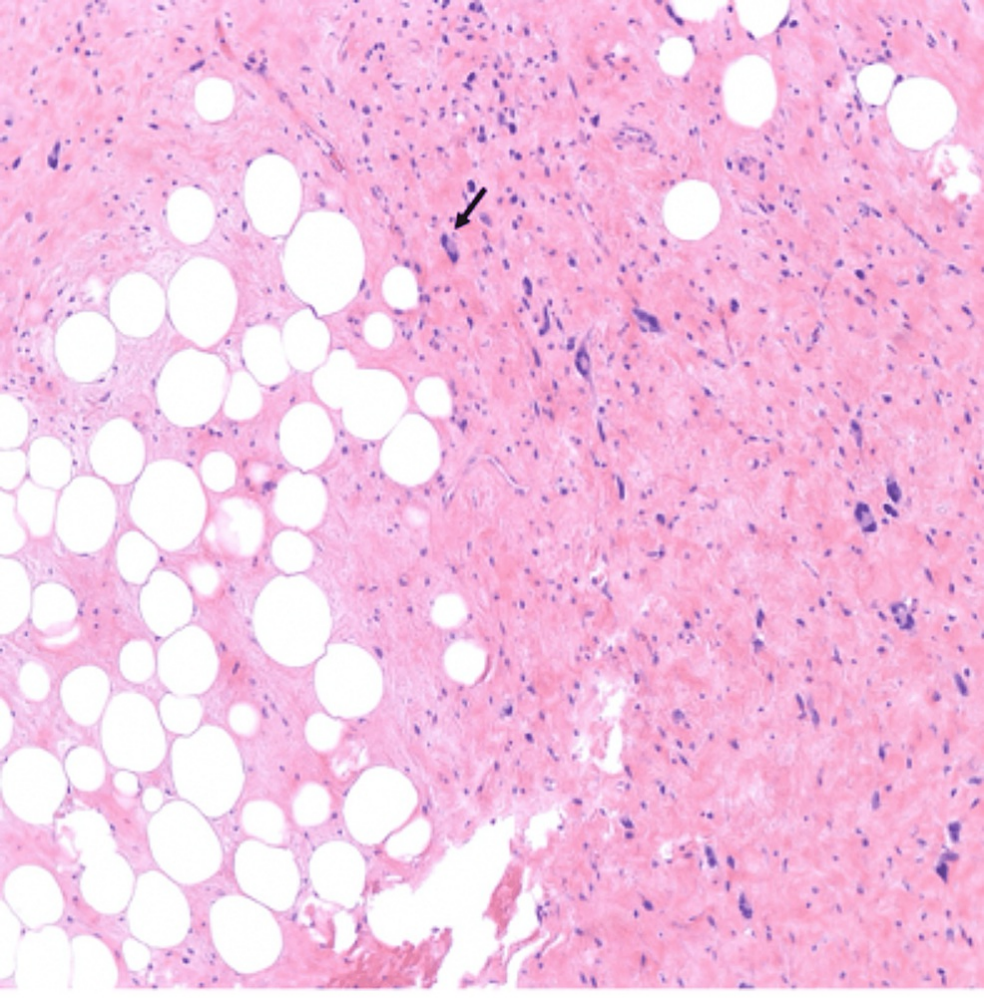
100x magnification H&E stain microscopic image showing variably sized mature adipocytes with bands of fibrotic stroma containing spindle cells with enlarged, hyperchromatic nuclei (arrow) H&E: Hematoxylin and Eosin

Hematoxylin & eosin (H&E) microscopic images show WDL on the right and kidney tissue on the left. The tumor abuts but does not invade adjacent kidney tissue (Figure 4). H&E microscopic imaging also showed variably sized mature adipocytes with bands of fibrotic stroma containing spindle cells with enlarged, hyperchromatic nuclei (Figure 5). These histopathology findings indicate that the tumor is a WDL, as opposed to an AML.

The patient's post-operative course was uncomplicated and the patient was discharged on day five. At the follow-up visit one week after discharge, the patient was found to be recovering well and was pending follow-up imaging. 

## Discussion

Soft tissue sarcomas account for less than 1% of malignancies in adults, and around 10-15% of soft tissue sarcomas in adults occur in the retroperitoneal space [4]. Of all the soft tissue sarcoma subtypes, liposarcoma is the most common, accounting for approximately 20% of all soft tissue sarcomas in adults [5]. Retroperitoneal liposarcomas are the most common primary retroperitoneal neoplasm. These tumors typically affect middle-aged patients, and the main treatment is surgical resection. Despite complete surgical resection, these patients have a poor prognosis [3].

This case reports a liposarcoma that was treated with SAE therapy due to radiologic tumor characteristics mimicking an angiomyolipoma [4,5,6]. The clinical diagnosis of retroperitoneal liposarcomas is difficult because these tumors are typically asymptomatic until they become large enough to compress neighboring abdominal organs. These tumors can be painless or present with abdominal pain, anorexia, weight loss, and nausea.

In the literature, there are some cases of renal AMLs mimicking WDLs due to similarities in radiographic imaging [6,7,8,9]. It is important to correctly diagnose these entities, as AMLs and WDLs have different treatment modalities. While AMLs have multiple treatment options, the management of liposarcomas is mainly surgical. Additionally, the clinical course of these two entities is very different. Renal AMLs are benign; however, the biggest clinical concern with these tumors is the risk of retroperitoneal hemorrhage, which can be life-threatening. Indications for treating these tumors are a size of ≥4 cm and the presence of symptoms regardless of size [9].

CT and MRI are the standard imaging tools used for the evaluation of retroperitoneal liposarcomas and can help distinguish the composition, relationship to neighboring organs, size, and enhancement [1]. Typically, AMLs and WDLs are both large fat-containing lesions which make them difficult to distinguish on MRI and CT, however, there are three distinctions that can be made in imaging to help identify the lesion correctly [8,9,10,11]. Should the observed mass be identified as an AML, a CT scan will reveal defects in the renal parenchymal structure, attributed to its expansion within the renal parenchyma. Conversely, in the case of a WDL, there is no resultant parenchymal irregularity, given their general non-invasive propensity towards the renal parenchyma. Moreover, it is noteworthy that WDLs, characterized by their avascular nature, will not exhibit conspicuous hypertrophied vasculature under contrast-enhanced CT imaging [8]. This stands in contrast to AMLs, where vascularization is a common hallmark, consequently presenting with discernible dilated vascular conduits apparent on CT scans. Other features that can be identified on CT or MRI, include a “beak sign” and “embedded organ sign” that renal AMLs have been noted to have [11]. 

Although CT and MRI imaging are used to evaluate these masses, the final diagnosis is determined using pathological analysis. There are five different subtypes of liposarcomas, which include well-differentiated, dedifferentiated, myxoid, round cell, and pleomorphic. A negative prognostic factor for patients with retroperitoneal liposarcomas is a high histological grade. The subtypes that are classified as high-grade are round cell, dedifferentiated, and pleomorphic, while myxoid and well-differentiated are considered low-grade [11]. The two most common subtypes are well-differentiated and dedifferentiated. Histological analysis of WDLs reveals a composition predominantly constituted by mature adipocytes, with varying amounts of lipoblasts. Lipoblasts are the defining cells of liposarcomas and are usually sizable, containing multiple vacuoles, and characterized by scalloped nuclei and hyperchromatic atypical nuclei [1]. In contrast, histological analysis of renal AMLs elucidates a histological profile characterized by a triphasic pattern, featuring myoid spindle cells, fully developed adipose tissue, and distorted thick-walled blood vessels lacking elastic lamina. These smooth muscle cells manifest a polymorphic nature ranging from epithelioid to pleomorphic morphologies [11].

In McGrath et al., it was reported that complete resection of liposarcomas can be carried out in up to 70% of cases when the tumor was >10 cm [12]. Retroperitoneal liposarcomas typically require a more aggressive surgical approach and at times multiorgan resection is necessary. After complete resection of WDLs, the five-year survival rate increases from 16.7 to 58%. However, the risk of metastasis is low in these tumors [3]. Radiation therapy can be used for high-grade liposarcomas, but the toxic effect can limit this therapeutic option to be used as a primary modality [13,14]. Research shows limited benefits for adjuvant chemotherapy in the treatment of WDL. Therefore, complete resection is the standard treatment that leads to the best outcomes. 

In the present case, the tumor was identified as a renal AML and treated with SAE without prior urologic consultation. If a core-needle biopsy had been performed this could have provided additional information in guiding the diagnosis and avoided the patient undergoing unnecessary procedures. 

## Conclusions

We report a case of a large retroperitoneal WDL that mimicked a renal AML on radiographic imaging. The clinical diagnosis of retroperitoneal liposarcomas is difficult since these tumors are typically asymptomatic until they become large enough to compress neighboring abdominal organs. It is important to correctly diagnose these entities as AML and WDLs because they require different treatment modalities. Given the similarities between renal AML and WDLs, a high index of suspicion is needed for the diagnosis. A core-needle biopsy could be helpful in the diagnosis of WDLs. This screening method could have provided additional information to guide the diagnosis and helped the patient avoid unnecessary procedures.
